# Supplementary material for: Benzodiazepines Drive Alteration of Chromatin at the Integrated HIV-1 LTR
Source: Viruses. 2020 Feb 9;12(2):191. doi: 10.3390/v12020191 (PMC7077212; doi:10.3390/v12020191)
Supplement: Supplementary file 1 [file viruses-12-00191-s001.pdf]

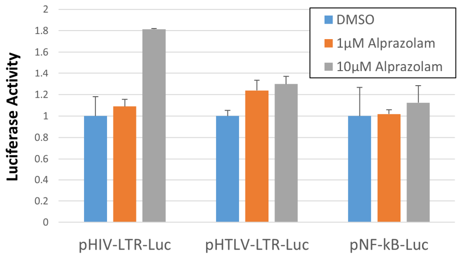

Supplementary Figure S1. Responsiveness of non-HIV promoters to alprazolam. 293T cells were transfected with the indicated luciferase reporters (1μg). Twenty-four hours post-transfection, cells were treated with Alprazolam (10μM). Thirty hours post-treatment, promoter activity was measured by luciferase. Data from two independent experiments (comprising technical duplicates) were plotted as means ± SD.

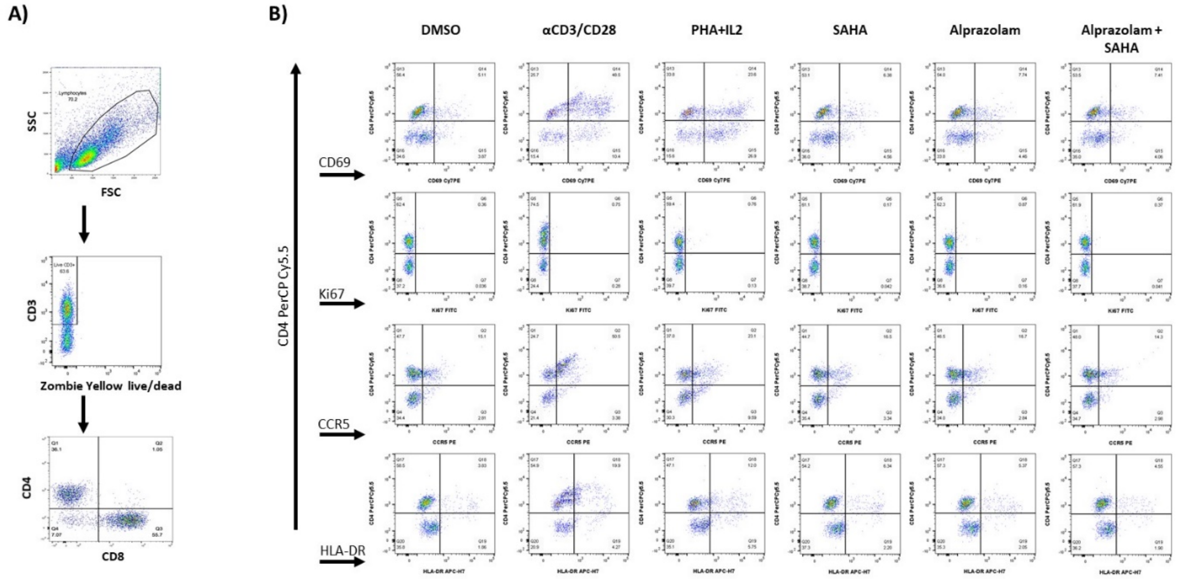

Supplementary Figure S2. Flow cytometric analysis of T-cell activation. (**a**) An example of the gating scheme used to identify live CD3+ T-cells and to differentiate between CD4+ and CD8+ cells. **(b)** PBMCs from a representative donor were stimulated for 24 hours with the indicated conditions before being stained with Zombie yellow live and dead staining and then stained for CD3, CD4, CD8, CD69, CCR5, HLA-DR and intracellular Ki67. Live T-cells (as indicated by the gating scheme shown in panel A) were then examined for the expression of each activation marker (X-axis) versus CD4 (Y-axis).
